# Tending the sick: Observations of epimeletic behavior in humpback whales towards conspecifics during entanglement events

**DOI:** 10.1371/journal.pone.0321284

**Published:** 2025-04-15

**Authors:** Rachel Cartwright, Ed Lyman, Amy Venema, Jens J. Currie, Stephanie H. Stack, Adam A. Pack, Lars Bejder, Martin Van Aswegen, Sadie K. Wright, Dorothy Horn

**Affiliations:** 1 The Keiki Koholā Project, Kihei, Hawaii, United States of America; 2 Department of Environmental Science and Resource Management, California State University Channel Islands, Camarillo, California, United States of America; 3 Hawaiian Islands Humpback Whale National Marine Sanctuary, Kihei, Hawai‘i, United States of America; 4 Pacific Whale Foundation, Wailuku, Hawai‘i, United States of America; 5 School of Environment and Science, Griffith University, Brisbane, Australia; 6 Psychology and Biology Departments, University of Hawai‘i at Hilo, Hawai‘i, United States of America; 7 The Dolphin Institute, Hilo, Hawai‘i, United States of America; 8 Marine Mammal Research Program, Hawai‘i Institute of Marine Biology, University of Hawai‘i at Manoa, Kaneohe, Hawai‘i, United States of America; 9 NOAA Fisheries, Alaska Protected Resources Division, Juneau, Alaska, United States of America; Soprintendenza Archeologia Belle Arti e Paesaggio Firenze Pistoia Prato, ITALY

## Abstract

Anthropogenic impacts on marine systems are increasing in frequency, geographic range and severity. While changes in climate will likely lead to the greatest impacts at the system-level, for marine megafauna, entanglement in marine debris also constitutes a pernicious threat. For baleen whales, in regions where high productivity and prolific fisheries overlap, entanglement is emerging as a component of their life history: In some of these regions, entanglement comprises the leading cause of serious injury and mortality. Additionally, up to 80% of whales carry scars indicative of entanglement, and associated declines in long-term health are reducing fecundity. Here, we describe behavioral traits seen in humpback whales during entanglement incidents. Specifically, we focus on reports of humpback whales that have remained in association with entangled whales during these incidents and apply the term “companion whales” in reference to these whales. Reports reviewed include a detailed account of a recent incident observed in Hawaiian waters, a compilation of 62 accounts of similar behavior extracted from 414 reports of entanglement events provided by regional entanglement response networks, and a series of six reports associated with whaling activities. The similarities between the current behavior of companion whales and behaviors observed during whaling activities suggest that this may be an example of behavioral plasticity, underscoring the expanding behavioral repertoire exhibited by baleen whales, and highlighting their potential resilience as they respond to the changing marine environment.

## Introduction

Anthropogenic impacts on marine systems are global in their reach [1], and currently increasing in both frequency and severity across all major oceans [2,3]. While climate change is by far the most ubiquitous issue impacting marine fauna [3–5], for baleen whales, entanglement in fishing gear, mooring lines and other marine debris also accounts for significant mortality [6,7]. In some regions, entanglement comprises the leading cause of death for large whales [8,9]; however, not all entanglements are lethal. When whales survive entanglements, long-lasting scars acquired during these events reveal valuable insights into the frequency, impacts and long-term outcomes for entangled whales. Typically, the frequency of entanglements peaks in regions where the range of large whales overlaps with highly productive fisheries [9]. For example, in the Gulf of St Lawrence, 85% of humpback whales (*Megaptera novaeangliae)*, 60% of blue whales (*Balaenoptera musculus),* and 50% of fin whales (*Balaenoptera physalus)* bear scars indicating previous entanglements [10]. Impacts associated with non-lethal entanglements range from impeded mobility and increased costs of travel [11], to declining body condition [12], reduced reproductive output and slowed recovery rates in rebounding populations [13]. Cumulatively, entanglement is now recognized as a prevalent and costly component of the life history of large whales [11], and death due to entanglement may be drawn out and distressing for whales that succumb in this way [14]. Comparing age classes, juvenile whales are more likely to become entangled, and to succumb to injuries resulting from these events [15].

Although recent data are somewhat sparse, regional studies focusing on non-lethal entanglement rates estimated annual entanglement rates of between 8 (northern South-east Alaska) and 25% (Gulf of Maine) [16,17]. Notably though, as whales in these studies were re-sighted with newly acquired entanglement scars, these studies confirm that many whales free themselves [16,17]. On the US West coast, an estimated 10% of entanglements are reported to disentanglement networks, under NOAA Fisheriesʻ Marine Mammal Health and Stranding Response Program [18]. When feasible, authorized disentanglement teams respond on the water, and may attempt to free whales from life-threatening entanglements. Success rates for these teams are currently approaching 50% [18]. The entanglement response teams on the West Coast, along with similar groups in the Pacific Islands, and in Alaska, also gather information that may enhance our understanding of the dynamics of these traumatic and life-threatening events. Such information will potentially further inform disentanglement efforts. They may also serve to mitigate threats both to whales and personnel, and to increase future rates of success. Extending these efforts to better understand the behavior and responses in associated, but uninjured conspecifics will further support these goals.

Epimeletic behavior is defined as care or attention provided by a healthy individual towards another individual that is distressed. Typically, this also encompasses behavior directed toward an animal once it is deceased [19]. Most reports of epimeletic behavior in animals reference mammals, and those involving charismatic mammals often garner a great deal of public attention. High profile examples include African elephants (*Loxodonta africana)* gathering around an ailing matriarch [20], and mountain gorillas (*Gorilla beringei beringei*) providing care for an injured juvenile orphaned during an attack by poachers [21,22]. In reality though, epimeletic behavior has been observed in a wide range of mammals, ranging from Barbary macaques, (*Macaca sylvanus)* [23], wolves (*Canis lupus)* [24], manatees (*Trichechus manatus)* [25], giraffes (*Giraffa camelopardalis*) [26], and hippos (*Hippopotamus amphibius)* [27], to lab-raised rodents [28].

Within marine mammals, epimeletic behavior to date has most commonly been reported among toothed whales [29]. Nurturant epimeletic behavior, directed at offspring or younger individuals, is most frequently seen. Maternal behaviors include supporting calves at the surface before or after the calf’s demise, and carrying recently deceased calves during travel. Documented examples include Atlantic spotted dolphins (*Stenella frontalis)* [30], Risso’s dolphin, (*Grampus griseus)* [31], and killer whales, (*Orcinus orca)* [32]. Related members of the family group and other affiliated group members may also display epimeletic behavior, assisting either the mother (in killer whales, [32]), or the ailing calf (in Indo-Pacific humpback dolphins (*Sousa chinensis)* [33]). By comparison, succorant epimeletic behavior, directed at adult conspecifics, is rarely observed [29] and typically, this comprises highly coordinated responses within affiliated groups. Examples include an incident wherein a group of 10 to 12 long-beaked common dolphin (*Delphinus capensis)* gathered around and supported a distressed conspecific at the surface prior to its demise [34], and in somewhat similar circumstances, a group of Atlantic bottlenose dolphins (*Tursiops truncatus*) formed a raft to support a distressed individual at the surface [35].

In contrast, detailed accounts of possible epimeletic behavior among baleen whales in recent literature comprise of a single incident [36]. This incident involved a group of several humpback whales engaged in a bout of competitive behavior in Hawaiian waters. In this context, competitive behaviors typically involve two or more males jockeying, at times aggressively, for access to a female [37–39]. Following the death of a male whale in one of these groups, several whales gathered around the deceased whale. One male remained with the dead whale for over four hours, alternately holding a stationary position below the whale, surfacing adjacent to it, displaying its penis and grasping the dead whale with its pectoral fins from a ventral position [36]. While the authors considered the possibility that this was an example of epimeletic behavior, Pack et al. [36] interpreted these events as predominantly sexual in nature. Recent observations by Stack et al. [40] provide an additional detailed description of sexual behavior between two male humpback whales, and serve to confirm this interpretation.

Other published accounts of epimeletic behavior in baleen whales can be found in earlier literature; these reports exclusively comprise of observations during whaling activities. Caldwell and Caldwell [41] summarize a selection of eye-witness accounts, describing behaviors that were both nurturant and succorant in nature: Mothers displayed a tendency to stay beside their injured calves, while uninjured conspecific adults accompanied and supported other captured adult whales. Notably, accounts involved a range of different species, including gray whales (*Eschrichtius robustus),* North Atlantic right whales (*Eubalaena glacialis)*, bowhead whales (*Balaena mysticetus)*, blue whales and humpback whales.

In this study, we focus specifically on humpback whales. As migratory mysticetes, humpback whales move annually between high latitude feeding grounds, where they exploit rich prey resources, and low latitude, nutrient poor breeding grounds [42,43], where reduced predation pressure benefits newborn and young calves [44,45]. The global population of humpback whales today represents a resurging population. Commercial whaling activities reduced humpback whale numbers to a global low by the mid-1960’s, but with the cessation of whaling, numbers have rebounded. In 2016, the global population was reclassified into 14 distinct population segments (DPS) [46]. Ten of these segments, including the Hawai‘i DPS,were deemed no longer at risk [47]. In some regions, this recovery serves as a conservation success story [48]. However, in other regions, such as the North Pacific, climatic conditions have brought new challenges [49], leading to pronounced fluctuations in population growth rates in recent years [50].

Here, we provide a first-hand account of epimeletic behavior among humpback whales in Hawaiian waters, alongside a review of archived accounts of epimeletic behavior documented during authorized disentanglement responses to humpback whales, reported in Hawaiian and Alaska waters, under NOAA’s Marine Mammal Health and Stranding program (MMHSRP). We use these details to discern the range and current frequency of epimeletic behavior among humpback whales during entanglement events. We then compare the behavior seen on recent occasions with accounts of similar behavior reported in the whaling literature. Finally, we discuss the evolutionary forces that may have shaped the frequency and expression of this type of behavior among baleen whales today. Taken cumulatively, these accounts serve to increase our understanding of the behavioral responses of marine mammals during entanglement events, and contribute towards the on-going development of effective mitigation strategies and response to the ever-increasing threat of entanglement for today’s marine mammals.

## Method

For this study, data were collated from three sources: (1) During first-hand field observations, describing a specific incident in Hawaiian waters between 9 Mar 2021 and 11 Mar 2021, (2) from regional entanglement reports, covering two regional networks (Hawai‘i and Alaska) between 1 Jan 2001 and 31 Dec 2023, and (3) from published literature, obtained during a comprehensive literature search using subject-specific keywords.

### Field observations

Ethics Statement: All activities were conducted under research permits (NMFS NOAA permit #22750, NOAA MMHSRP permit # 18786, NMFS NOAA #21321), and in adherence to all guidelines laid out in these permits. Full details of the precise research protocols used in this study were reviewed by the Office of Protected Species, prior to issuance of the above research permits. Inherent in this review, is the requirement that every effort be made to minimize any impact on animals during research activities. As this detailed and extensive review had been conducted by experts in this field, further ethical review by the co-operating institution, California State University Channel Islands was not required. All research protocols additionally comply with the Endangered Species Act (1973) and the Marine Mammal Protection Act (1972) (https://apps.nmfs.noaa.gov/docs_cfm/laws_and_regulations.cfm).

**Fig 1:**
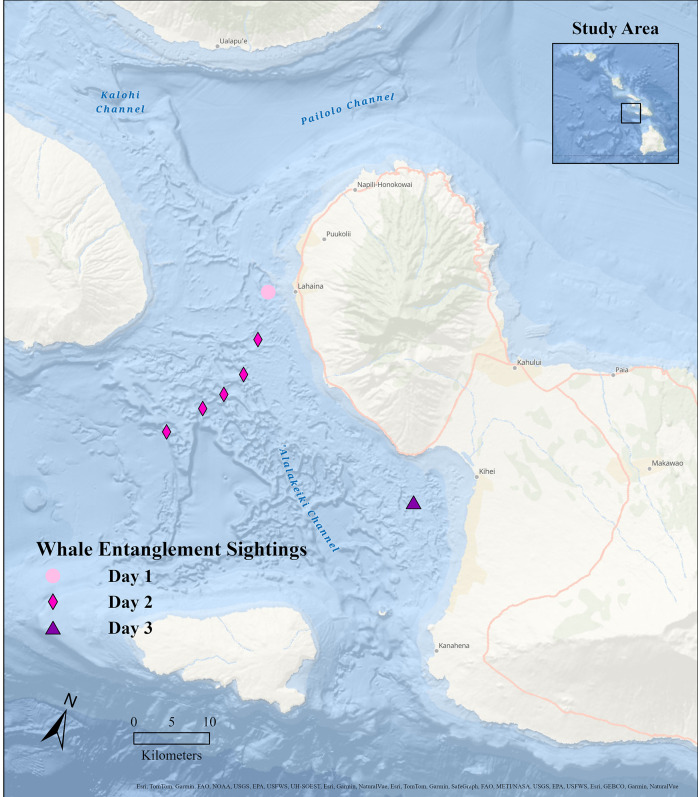
Study area, showing sightings of an entangled whale and two companion whales in the Au‘Au Channel, Maui, Hawai‘i, between 9 Mar and 11 Mar, 2021. Credits: Esri, Garmin, GEBCO, NOAA NGDC, and other contributors.

Field observations took place in the Au‘ Au Channel, West Maui, Hawai‘i (20.8520 N, 156.8400 W; Fig 1), between 9 Mar and 11 Mar 2021. The Au‘Au Channel, which lies between the islands of Maui and Lāna‘i, is characterized by gently sloping shoreline gradients, with maximum water depths of approximately 150 m [51]. The focal entangled humpback whale observed here was first reported to the Hawaiian Islands Humpback Whale National Marine Sanctuary (HIHWNMS) on 13 Feb 2021 by private citizens (Robert and Ellen Raimo). The HIHWNMS coordinates large whale entanglement response efforts in the waters around Hawai‘i under the oversight of NOAA Fisheries MMHSRP. Initially, the condition of the whale was reported as fair, slightly emaciated, with small patches of cyamids distributed over the portion of the body visible at the surface, and with visible entangling lines, however at this stage the extent of the entanglement was not known. On 9 Mar 2021, the entangled whale was opportunistically re-sighted earlier in the day by tour vessels and with subsequent monitoring support from members of the on-water community, the sanctuary-led team was able to respond and fully document the entanglement (described below). By this time, the animal’s condition had deteriorated; experienced entanglement team members described the animal as being in very poor condition, clearly emaciated and mostly covered in cyamids. The team was able to remove all but the embedded line in the mouth along with a small amount of trailing gear. On 10 Mar 2021, the still-partially entangled whale was observed with two “companion” whales (see definition below), and on 11 Mar 2021, the partially entangled whale was re-sighted alone. This was the last confirmed sighting of the entangled whale.

Throughout all periods of response and observation, extensive efforts were made to minimize disturbance and ensure that the response, observation and documentation of the whales involved did not add to their distress. Surface, aerial and underwater observations were limited to short intermittent periods, interspersed between periods where research vessel(s) would stand off and remain at a distance of between 100–300 m from the whale(s) for extended periods of time.

During observations, photographic images from the surface were gathered using digital cameras with zoom lenses (e.g., a Canon 6D DLSR camera equipped with a 300 mm lens and a Canon 7D DSLR camera equipped with 100–400 mm lens). Unoccupied aerial systems (UAS; drone) video footage and still imagery data were obtained using a DJI Phantom 4 and a DJI Inspire 2 (the latter was equipped with a custom-fitted altimeter and an Olympus 25 mm f1.8 rectilinear lens with no distortion). Unoccupied aerial system (UAS) flights were operated by Part-107 authorized pilots in compliance with standards set by the Federal Aviation Administration. Underwater video footage and still imagery were recorded using a Canon Mark 5 DLSR, a high resolution RED underwater camera, both in Gates housings, and a pole mounted GoPro camera. Location data were recorded on an iPad running navigational software (INavx), and on camera-integrated GPS units. As part of their disentanglement efforts, crews also employed helmet- and pole-mounted GoPro cameras to document the animal and the nature of its entanglement.

### Regional entanglement reports

We reviewed reports and associated photo-documentation of confirmed entanglement cases involving humpback whales reported in regional records covering Hawaiian and Alaska waters, between 1 Jan 2001 and 31 Dec 2023. All incidents that included accounts of the presence and behavior of additional whales were compiled for further analysis and review. Some, but not all of these reports were provided to a member-only North Pacific Large Whale Disentanglement Response Network and some, but not all included photo documentation.

### Published literature

We systematically searched the published literature, using Google Scholar and Web of Science, for accounts of the behavior of baleen whales during entanglement events, and for any references to epimeletic or care-giving behavior in baleen whales. Keywords were used in combinations; whale_entanglement _behavior; whale_epimeletic_behavior and whale_caregiving. For each search term, the first 50 results obtained were reviewed. The literature search was further enhanced by utilizing an AI-enabled search engine (scite.ai [52]) to identify any additional, relevant published records. Details of any possible incidents involving epimeletic behavior among baleen whales were then compiled for further review and analysis. All references found were sourced back to first person/ first published accounts, wherever possible. These accounts were then sub-divided into accounts where the events seen were not associated with commercial whaling activities and where the events and behaviors reported were observed during commercial whaling activities.

### Data analysis

We compiled an ethogram, based on published definitions of epimeletic behavior that have been in use across the range of published literature in this field for more than 50 years [41,53]. The ethogram provides detailed descriptions and classifications of the full range of behavior that has previously been recognized as epimeletic, ranging from simple observance and mimicry, to the provision of active assistance. This follows the Russian Doll model, first proposed by de Waal [54] which endorses the inclusion of the full range of potentially responsive behavior. This ethogram was subsequently used to identify and classify any potentially epimeletic behaviors from each of the three specific sources.

In the descriptions and accounts of the events provided here, we use the term “associated” to describe whales that were traveling with, but did not show any response, towards a distressed or entangled whale. The term “companion whale” is used in reference to associated whales that changed their behavior in some way to maintain the affiliation (i.e., by altering typical or expected travel patterns) such that they remained in association with an entangled or distressed whale. Some, but not all of these companion whales, then potentially engaged in additional epimeletic behavior directed towards the afflicted whale. These additional behaviors were also defined and described, using the definitions provided in Table 1.

**Table 1:** Ethogram of epimeletic behaviors.

Behavior	Description
Standing by	Responding animal(s) remain in or approach the vicinity of the sick, injured or distressed animal, but do not offer evident aid or assistance. Equivalent terms used in whaling literature include “heaving to”, “laying to” or “bringing to a slow” *.
Excitement/ assistance	Responding animal(s) approach the sick, injured or distressed animal and exhibit behaviors associated with hyper-excitability or distress. These include circling of the injured animal, interception of vessels associated with the trauma, removal of the injured animal away from a presumed source of danger, and biting and attacking the source of danger, such as predators, fishery or capture vessels *. Assistance behavior, when the distressed animal derives benefit or assistance as a result of the actions of the responding animal, was included as a sub-category of this type of behavior **.
Supporting	Responding animal(s) actively support the sick, injured or distressed animal at the surface, potentially facilitating breathing by the injured or distressed animal *,**.

* [41].

** [53].

It should be noted that these summaries are not presented as an inclusive list of all epimeletic behaviors in the different arenas or contexts (i.e., during entanglement events in these regions, or during whaling activities). Rather these summaries provide a representative compilation of accounts of a specific component of whale behavior observed and/or recorded during these incidents, events and activities. This is particularly relevant to details derived from regional entanglement networks, as there was no mandate or requirement for reports to include the behavior of other whales observed during entanglement events. Therefore, this summary is not an exhaustive list of all epimeletic behaviors observed during entanglement response, and should be considered an under- or minimum estimate.

### Field observations

For analysis, video footage and still images of the entangled whale obtained in the field between 9 Mar 2021 and 11 Mar 2021 (including surface, aerial and underwater imagery) were compiled into a single chronological sequence, using the embedded time stamps. This was then reviewed, in conjunction with field notes, and instances of epimeletic behavior (as described in the ethogram provided in Table 1) were identified.

Surface and underwater images of the ventral surface of the tail flukes of both the entangled and accompanying whales were compiled to use for identification purposes. The ventral surface of a humpback whales’ tail flukes, along with the pattern of the trailing edge, are unique to each individual and therefore can be used for identification [55]. These images were submitted to Happywhale, an online digital database housing humpback whale fluke identifications. This database can be used to identify the individual whales involved, using Happywhale algorithms (Humpback whale automated image recognition: https://rdcu.be/cCOtw) and the archived dataset housed by Happywhale (https://rdcu.be/dfdPF). Where possible, the sex of each whale was established either through genetic analysis of skin/blubber biopsy samples, where such details had been archived with Happywhale, or through review of underwater imagery for detection of the hemispheric lobe, unique to females and located just caudal to the genital slit [56]. All relevant information sourced through Happywhale was verified with the individual contributors, and appropriate permission for use was obtained.

### Regional entanglement reports

Reports provided between 1 Jan 2001 and 31 Dec 2023, to NOAAʻs Marine Mammal Health and Stranding Response Program data archives were reviewed. These records are curated by the Hawaiian Islands Humpback Whale National Marine Sanctuary for the Hawaiian region and NOAA Fisheries Protected Resources Division for the Alaska Region. In all instances where other whales were associated with an entangled whale, the case was selected for inclusion in the study. The details provided were then reviewed to determine if the behavior described for the associated whales reached the threshold for epimeletic behavior, as defined in Table 1. However, as mentioned above, while the behaviors of other whales were often included in the reports, there was no specific requirement for responders to report the behavior of other whales in the area during entanglement responses. Consequently, this analysis of records in the database represents a summary of the reported occurrences, but not the absence of these behaviors.

### Published literature

Published accounts from the results of the literature search were compiled, reviewed and evaluated using sources that were as close to first-hand accounts as possible. Epimeletic behavior was identified based on the definitions laid out in the ethogram in Table 1.

## Results

### Field observations

The following observations were made between 9 Mar 2021 and 11 Mar 2021. In order to reduce the risk of disturbance, observations were intermittent and many periods of observation comprised of surface-only monitoring from a distance of 300 m or more. Consequently, while the timing of observations and descriptions of all behaviors observed are reported here, this account likely does not include every instance or accurate durations of each behavior. A general narrative is provided below:

**9 Mar 2021**
**Location: 20.8673, 156.7045**
**Water depth: 49 m, distance from shore: 2.41km**
**Relative local location: East side of Au‘Au Channel, adjacent to Lāhainā Harbor.**

**Fig 2:**
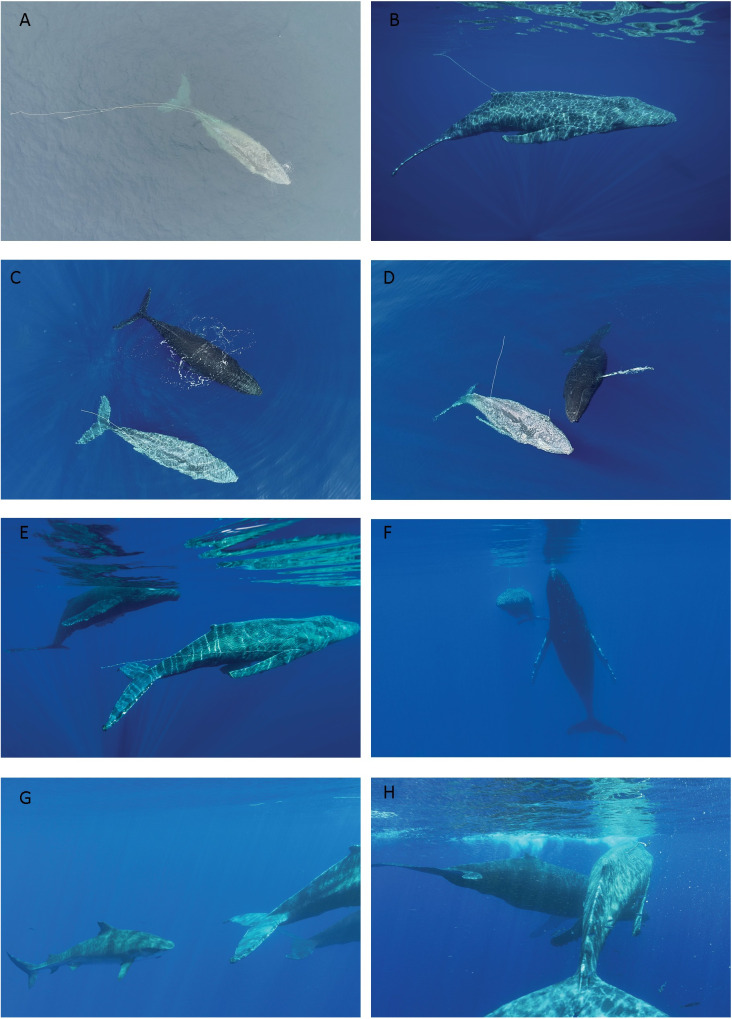
Aerial and underwater images of an entangled whale and a companion whale, taken on 9 Mar 2021 and 10 Mar 2021 in Maui waters. Image A shows the entangled whale (EW) alone on 9 Mar 2021 prior to any disentanglement assistance. Images B through H were taken on 10 Mar 2021, between 10 am and 5 pm. Image B shows EW on the morning of 10 Mar 2021, with a single line, passing through the mouth of the whale, remaining. In images C to H, the two whales in view are EW and H2. Images taken during research activities authorized under NMFS NOAA permit# 22750, and NOAA MMHSRP permit# 18786. Photo credit: Dan Cesere (underwater image B and E), Didier Noirot (underwater video, used to extract images F, G and H).

On 9 Mar 2021, at ~ 12.40 pm, local whale-watching vessels, (*Teralani* and *Wahine Hana*) first reported an entangled whale (hereafter referred to as EW) to NOAA’s Marine Wildlife Hotline, as the sighting was within sanctuary waters. The sanctuary research and response vessel, *R/V Koholā*, carrying a survey team, arrived at the location of EW at approximately 2 pm and provided an initial assessment, determining that the entanglement comprised of one line that passed through EW’s mouth, and was then wrapped three times around the animal’s left pectoral flipper, with a pair of lines trailing 15 m behind the whale. The whale’s condition was assessed as poor; the skin was light in color and roughened, the whale was covered in cyamids, and very emaciated (Fig 2A).

The sanctuary boat was joined by researchers from the Keiki Koholā Project (KKP) who assisted with the initial assessment, by providing approved UAS assessment and documentation. These first responders were later joined by trained and authorized responders from the West Maui Rapid Response team and HIHWNMS onboard the West Maui Response teamʻs dedicated research and response vessel, *Aloha Kai*, provided by Ultimate Whale Watch, to begin disentanglement efforts. At this point, KKP and *Koholā,* provided safety and assessment support. On the first approach, the response team removed approximately 10 m of trailing gear. On subsequent approaches an additional 12.5 m of line was removed. The team could not pull the line out of EW’s mouth as it remained too deeply embedded at the back of the mouth. At this time, all the wraps from the left pectoral flipper had been removed, and less than ~ 8 m of line remained on the animal. However, as EW became less cooperative, a decision was made to stand down.

No other whales were seen associated with or in the immediate vicinity of the EW during the initial observations and disentanglement of the whale on 9 Mar 2021. The response team terminated their efforts to assist the whale at 5.51 pm, at which point EW was swimming slowly, taking short-duration, shallow dives, mostly persisting at the surface and moving on a southerly heading.

**10 Mar 2021**
**Location: 20.8230, 156.6967**
**Water depth: 65 m, distance from shore: 5.01km**
**Relative local location: East side of Au‘Au Channel, slightly south of Launiopoko**

On 10 Mar 2021, at ~ 9.40 am, the entangled whale (EW) was encountered by KKP researchers (Fig 2 B). Surface and aerial observations began; full details of the behavior of all whales seen from this point on, over the course of the day, are provided in Table 2.

**Table 2:** Timeline of observations of an entangled whale and companion whales in Maui waters.

Date	Time	Observations	Epimeletic Behavior
10 Mar 2021	9.40 am	EW resighted, alone and swimming slowly south. Making short dives, resting at the surface, some surface behavior (several peduncle throws).	Absent
	10.48 am	Two whales sighted in the vicinity of EW. Both approach, one (H1) tail slaps beside the entangled whale, then leaves the area. The second whale (H2) remains beside EW, as they continue south.	Present – Standing by
	11.01 am	H2 swimming beside EW, observed together throughout aerial observation period lasting 11 minutes. H2 performs repeated head rises beside EW.	Present – Standing by
	11.15 am	EW resting at surface, moves into slow swim and dives for a short period, leaving the surface and maintaining a position 3 m down in the water column for a brief period. H2 is in position behind EW, around 2 m off her fluke at a lower depth. H2 moves off slightly, tiger sharks approach EW and appear to bite her fluke. Blood visible in the water. H2 reappears, performs head rises, then maintains a vertical position in the water, level with the rostrum of EW for a period of three to four minutes. At 12.15 pm, H2 surfaces to one side, dives below and lifts EW out of the water. At this point, the pair are moving slowly through the water. They approach and remain in close proximity to the research vessel for several minutes.	Present – Standing by, Assistance Present – Supportive
	12.26 pm	H2 observed swimming repeatedly in circles around EW. Swims below and to the rear of EW. Sharks can be seen dispersing during this behavior. H2 seen swimming directly below EW and repeatedly lifting EW out of the water over a two-minute period between 12:34 and 12:36 pm.	Present – Standing by, Assistance, Supportive
	1:43 pm	EW and H2 still together. Scars, or possibly lice, on the ventral surface of H2 now visible. H2 consistently repeats the circling behavior, and adds tail swishes and body rolls, with pectoral fins extended towards EW.	Present – Standing by, Assistance
	1:49 pm	H2 supporting EW at the surface	Present –Supportive
	1:54 pm	H2 swims out in front of EW, performs a sequence of 3 body rolls, then returns to the side of EW. This is repeated multiple times.	Present – Standing by
	3.00 pm	H2 repeatedly circling around EW, then positioned upright in water. With sharks in view, H2 makes contact and lifts EW. Subsequently, H2 lays head-to-head, almost touching and then relocates to behind and below the fluke of EW. EW makes a distinct dive, leaving the surface for a period of approximately 2 minutes.	Present – Standing by, Assistance, Supportive
	4.24 pm	H2 still alongside EW. The pair are maintaining the same speed and heading, slightly southwest.	Present – Standing by
	4.45 pm	H2 swimming ahead of EW by 2 body lengths, returns and swims rapidly alongside, then moves ahead again.	Present – Standing by
	5.15 pm	H2 breaches, at a distance of 2–3 body lengths ahead of EW	Absent
	6.15 pm	Final sighting: EW is unaccompanied by other whales. Swimming slowly, trailed by 6 to 7 sharks.	Absent

Intermittent observations continued over the course of the day, however the timeline for these observations is not continuous, as the research vessel stood back at a substantial distance from the whales for most of this time. Two other whales were sighted in the immediate area of EW at 10.48 am. The first whale, hereafter referred to as H1, remained only briefly beside EW (less than 5 minutes). The second whale (hereafter H2) then took over that position and remained directly beside or within the vicinity of EW for the following 5 + hours. Aerial observations at 11 am revealed approximately 10 to 12 tiger sharks (*Galeocerdo cuvier*) following EW and H2. Throughout the next five hours, H2 mostly remained in close proximity to EW, placing itself alongside and matching the slow swim speed of EW (Fig 2 C), remaining stationary at the surface, with its rostrum closely aligned to EW (Fig 2 D), in a stationary position around a ½  body length behind EW, or circling rapidly around EW, (visible from the air and underwater). This circling behavior had the effect of dispersing the numerous tiger sharks that were surrounding the pair. Other behaviors demonstrated by H2 included slow rolling alongside EW with the pectoral fins extended, vertical head rises (Fig 2 F), and supporting EW at the surface (Fig 2 H). H2 also made intermittent physical contact, with a single pectoral fin laid over the upper body of EW. The separation of the pair began around 4.30 pm, with H2 moving progressively further off from EW. The final sighting of H2 occurred at approximately 5.15 pm, and at the final sighting of EW, at 6.15 pm, EW was unaccompanied.

Reviewing these behaviors against the definitions provided in Table 1, the behavior of both companion whales (H1 and H2) met the definitions of epimeletic behavior, with H1 standing by briefly and H2 providing intermittent assistive and supporting behavior over a 5-hour period (between 11.15 am and 4.30 pm).

**11 Mar 2021, 1.45 pm**
**Location: 20.74143, − 156.50174**
**Water depth: 54 m, distance from shore: 4.43 km**
**Relative local location: Mā**ʻ**alaea Bay**

On 11 Mar 2021, at approximately 1:48PM, a collaboration of researchers on-board the Pacific Whale Foundation vessel *R/V Ocean Protector* resighted the entangled whale in a region 22.2 km to the south of the sightings on the previous two days. At this point, EW was resting just below the surface, and coming up to breathe every few minutes. EW was alone and at least three tiger sharks were visible in the area just behind the whale. EW alternated between a slow swim and stationary/resting behaviors throughout the observation period. No other whales were present during the encounter. Additional health assessment details, including UAS body condition measurements (for methods, see Christiansen et al [57,58]), and GoPro underwater footage were obtained. Observations continued for 36 minutes. At last sighting, the entangled whale was still alone and headed on an easterly course, towards the shoreline of South Maui.

### Fluke identification, sighting histories and morphometrics

Fluke identification images were obtained for all three whales, and submitted to Happywhale, which provided sighting histories and sex details (see Table 3). Using aerial photogrammetry methods developed by Christiansen [57,58], the entangled whale (EW) was confirmed to be a small juvenile (length 10.3 m; estimated age 3 – 4 years), with an estimated body volume of 12.3 m^3^. Body condition of the EW was poor, with EW’s body volume 21.5% lower than predicted for North Pacific humpback whales of the same length [59].

**Table 3:** Sighting histories of humpback whales involved in epimeletic behavior in Maui waters.

Role	Whale ID	Sex	First sighting	Most recent sighting	Min Age^*^	Additional details
EW	BCX1922	F	13 Jul 2019	11 Mar 2021	3	First sighting in Canadian waters. Sighted in Maui waters on 13 Feb 2021, already entangled
H1	SEAK-5566	M	16 Mar 1998	10 Aug 2022	26	First sighting was in Hawaiian waters, sighting on 10 Aug 2022 in Southeast Alaska
H2	HW-MN0441350	M	3 Mar 2005	11 Mar 2021	19	First sighted in Philippine Sea. Resighted on 11 Mar 2021 in a group > 4 whales, including a mother-calf pair, in Hawaiian waters.

EW – Entangled whale.

H1 – First companion whale sighted.

H2 – Second companion whale sighted.

*Age at the time of the event, based on age 3 at first sighting, as established by Happywhale.

Sighting details and Whale IDs retrieved from Happywhale.

Credits: Mark Sawyer, first sighting of BCX1922(EW); Mindy Huston, most recent sighting of SEAK-5566 (H1); Everlasting Nature of Asia, first sighting of HW-MN0441350 (H2); James Begeman, most recent sighting of HW-MN0441350 (H2). Other sightings by authors/ author organizations. Sex attributions: BCX1922 (EW) through review of underwater images by authors, SEAK-5566 (H1) through biopsy by A. Pack, HW-MN0441350 (H2) through biopsy conducted by Everlasting Nature of Asia/ Ogasawara Whale watching Association. All named individuals and organizations have granted permission for use of this data.

### Regional entanglement reports

In total, 414 additional accounts of entangled humpback whales were collected from regional entanglement reports for further review. These comprised of 154 events in Hawaiian waters and 260 events in Alaska waters. For Hawaiian waters, events occurred around the main Hawaiian Islands, in near- and offshore waters, up to several hundred miles from the island chain. The geographic range for Alaska waters extended from the Aleutian Islands, through the Gulf of Alaska and Southeast Alaska. All reported events occurred between 1 Jan 2001 and 31 Dec 2023. Although not a requirement of these reports, approximately 15% (62 of 414) of accounts mention other whales associated with or responding in some way to an entangled individual (S1 Table). Within these, 41 accounts were from Hawaiian waters and 21 were from Alaska.

Of these 62 events, 42% (26 of 62) involved mother and calf pairs. In 14 of these cases, the calf was entangled, and in 13 of those cases, the mother remained in association with the calf throughout the observed event. The one exception, where the mother left the calf, involved a whale called “Flame”, who is well known in the Alaska region. In this case, Flame was not seen during the period in which the calf received assistance from a local response team. However, she was seen later in the season accompanying her calf, with identification based on previously documented fluke patterns and dorsal fin profiles (Pers Observation; H. Pearson, University of Alaska Southeast).

**Fig 3:**
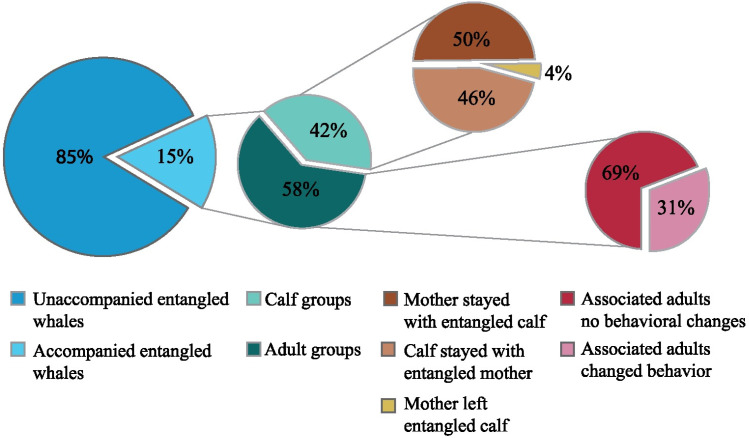
Instances of epimeletic behavior in entanglement events involving humpback whales reported to the North Pacific Large Whale Entanglement Network, between 1 Jan 2001 and 31 Dec 2023.

In nine cases involving mother and calf pairs, the mother was entangled, and in all these cases, the calf remained in close proximity to the mother throughout. In the three remaining cases, both the mother and calf were entangled. One case, in Hawaiian waters, involved a presumed yearlingThis presumption could have been based on body size, the time of the season, the whale's close association with an adult female, and/or the presence of large numbers of epiphytes, however the exact justification is not noted in the report. Three cases included male escort whales that remained in close association with the mother-calf pair. In two of these, the calf was entangled and in one case, the mother was entangled (Fig 3).

For the 36 cases that involved solely adult whales, in 25 cases the entangled whales were seen in groups, travelling with other whales. As observers did not report that the behaviors of the other whales were modified in any tangible way to accommodate the entangled whale, these events were classified as associated, but did not meet the criteria for companion whales. In the remaining 11 cases, associated whales changed their behavior in a way that accommodated the entangled whale (e.g., slowed their speed of travel to match the distressed whale, changed their direction of travel, relocated to shallow water, engaged in directed surface activity), therefore meeting the description of companion whales. In one protracted case, a slowly swimming pair in which one individual was entangled was seen together in multiple locations over a period of four days. The pair maintained a position close to the shoreline in all sightings.

In total, we identified 24 instances (out of the total of 62 cases), where the behavior(s) observed met the definitions of epimeletic behavior laid out in Table 1. Of these cases, 13 involved a nurturant response between mothers and offspring; the remaining 11 cases involved succorant responses, where adults responded to other adult whales (Fig 3, S1 Table). The most commonly observed response was standing by (17 of 24 cases), where whales altered their typical pattern, adopting slower travel speeds, unusual routes, and/or they made changes in course that resulted in the companion whale(s) remaining in close proximity to other injured whales. Among adult whales, two incidents were classified as assistive and one as supportive, while among mother-calf pairs, four cases were classified as assistive.

### Published literature

The combined results of the literature searches yielded numerous accounts relating to the behavior of toothed whales, but only a small number of accounts of epimeletic behavior in any species of baleen whales, including humpback whales. The first four were found using traditional search methods. These publications were also detected by the AI-enabled search engine, along with an additional recent publication, providing a total of five additional published accounts that warranted consideration. Two described the behavior of other humpback whales around entangled whales, two involved humpback whales responding to deceased animals, and the fifth study described accounts of humpback whale behavior reported during whaling activities.

The first related publication comprised a summary of entanglement reports between 1996 and 2021, in the waters of the Mexican Pacific and Baja California Peninsula [60]. These authors compiled accounts of 208 incidents, involving 218 large entangled whales. Of these, 187 (86%) involved humpback whales, and 3 of these included reports of behavioral responses seen in other adults. These responses were reported as displays of aggressive behavior directed to the rescue teams, such as charging, bubbling, trumpeting, and/or complicated maneuvers. Notably, the report specifically noted that these three incidents did not involve mothers, calves or associated escorts. No details of events that involved mothers, calves or escorts were provided. In the second account, Weinrich [61] describes a group of three humpback whales that approached a whale entangled in a gill net. The three other whales remained within 90 m of the entangled whale for around 20 minutes. The author reported coordinated trumpeting sounds, but no close approaches, or any means by which the whales provided any assistance to the entangled whale. The third case involved a deceased whale. As described here already [36] this was mediated by a male adult and directed towards a deceased male conspecific [36]. The fourth case also involved a deceased animal; Frediani, Black and Sharpe [62] described the behavior of two juvenile humpback whales interacting with a dead gray whale calf [61]. The fifth publication, a review paper, provided a range of accounts of baleen whale behavior around entangled whales, however all the events described occurred during whaling activities. Specifically, Caldwell and Caldwell [41] reference six events in which whaling crews provided firsthand accounts of companion whales responding to entangled whales during whaling activities. All six events were traced back to the first published accounts, with summaries of those first reports provided here (Table 4).

**Table 4:** Possible examples of epimeletic behavior in humpback whales observed during whaling activities.

Date	Location	Reported behavior	Epimeletic Behavior
^*^19 Aug 1934	Providence Bay, Bering Strait	Whaling crew reported that a wounded male took the ship in tow. Throughout the chase period, a large female remained beside the male and only left after the male was killed with a second shot.	Present – Standing by
^*^4 Oct 1934	Southwestern coastal waters, Chukchi Sea	Whaling crew reported pursuing three feeding humpback whales. After 30 minutes, the whales stopped and surrounded the vessel. The whalers shot one of the whales. The other two whales remained with the wounded whale. The whaling crew describe their behavior as excited. Some 20 minutes later the wounded whale was killed with a second shot. The other whales were not taken because the lines became tangled.	Present – Standing by, Assistance
^*^6 Sep 1934	Western coastal waters, Bering Sea	Whaling crew reported a pair of whales swimming together. When the male was wounded by the whalers, it took the ship in tow. The second whale (presumed female) stayed beside the wounded whale until it expired, some 20 minutes later.	Present – Standing by
^*^6 Sep 1934	Western coastal waters, Bering Sea	In a second incident on the same day, in the same region, the whalers chased a second pair, eventually wounding the presumed male. While the male towed the ship some distance, the second whale stayed beside throughout. When the wounded whale was eventually pulled into the ship, the second whale again remained beside it, until it was subsequently killed by the whaling crew. The second whale confirmed as female based on the presence of mammary glands.	Present – Standing by
^*^9 Sep1934	Providence Bay, Bering Strait	Whaling crew reported giving chase to a pair of whales. They shot one with a harpoon, and as that whale began to tow the ship, the second whale fled then returned to the side of the first whale. As the wounded whale was drawn into the ship, the second whale followed and was also killed.	Present – Standing by
^**^Zenkovich 1956		Crewman from a whaling vessel described a humpback supporting another injured animal at the surface for 40 minutes.	Present – Standing by, Supportive

*Taken from Tomilin [63].

**Taken from Zenkovich [64]; reported in Slipjer [65].

Caldwell and Caldwell [41] classified the behaviors observed in each of these cases as epimeletic behavior, as companion whales altered their behavior and remained with injured and distressed whales. The period of standing-by was prolonged in some cases. In three cases, the companion whale left when the original injured whale succumbed to their injuries (n = 3 of 5 cases). In two cases, the responding companion whales were subsequently captured and slaughtered. In the final case [65], the outcome was not specified.

## Discussion

While recent published accounts suggest that epimeletic behavior in cetaceans is mostly attributable to toothed whales (e.g., Bearzi et al. [29]), this study provides detailed descriptions of epimeletic behavior involving baleen whales, specifically, humpback whales. In toothed whales, epimeletic behavior is frequently directed towards deceased conspecifics [29,31–33]. Contrastingly, in this study focusing specifically on humpback whales, the majority of observations describe epimeletic behavior directed towards distressed and injured conspecifics.

An alternate explanation for this attention could be that these behaviors were sexual in nature. However, there were no reports of male genital displays during any of the observations reported here. Additionally, in descriptions of homosexual intercourse and contact between humpback whales that were recognized to be of a sexual nature [36,40], the male humpback whale initiating contact assumed a position above the targeted partner, used the pectoral fins to grasp the other whale from above, and the extruding penis was clearly visible. From the vantage points allowed throughout the aerial and underwater observations during the incident in Hawaiian waters, and during the additional entanglement events reviewed, no observations or visual evidence placed the whales in these relative positions or orientations, nor did any other evidence emerge to support the interpretation of the observed behaviors included in this study as being sexual in nature.

Rather, the behaviors described here fulfil the definitions of epimeletic behavior provided by Caldwell and Caldwell [41], and later modified by Connor and Norris [53]. Focusing on the nature of behaviors observed here, a striking resemblance emerges between the behavior of companion whales observed during recent entanglement events and the descriptions of the behavior of companion whales observed during whaling activities, gleaned from accounts describing events that took place over 75 years ago. Potentially, this may be an example of behavioral plasticity, whereby a previously acquired behavioral trait is modified in response to a new or novel environmental stimulus [66,67]. Commercial whaling in the North Pacific largely came to halt in the mid-seventies [68], so the frequency of whales encountering the lines of whaling operations would have dwindled to a state of rarity by this time. However, discarded fishing apparatus and abundant marine debris have been consistently present in our oceans [69], and may now serve as the stimulus for a comparable behavioral response [66,67]. This type of plasticity, whereby species’ respond to human-induced environmental changes through the plasticity of previously acquired behavioral traits, has been documented quite widely in recent years, both in a variety of different species, and across a broad selection of settings (see Wong and Candolin [70] for a detailed review).

Further characterizing the nature of epimeletic behavior, this type of behavior may be recognized as a form of affective empathy [71]. In affective empathy, an individual recognizes an emotional state, such as distress, in another individual without actually experiencing the stimuli themselves [72], and this extends to include sensory states, such as pain [73]. In the context presented here, the companion whale(s) are not impacted by the stimuli of the entanglement themselves.Rather, they are responding to the predicament of the entangled whale [74]. Interestingly, a proximate mechanism that could drive affective empathy in cetaceans has been identified: Studies of the neuroanatomy of mammals have established that affective empathic behavior is driven by the activation of specific spindle cells known as Von Economo neurons (VENs) in the frontal insular cortex (FIC) and in layer V of the anterior cingulate cortex (ACC) in the brain [75,76]. Studies of cetacean neuroanatomy have identified similarly placed spindle cells in the same locations in the brains of several species of cetaceans, including humpback whales [77,78]. Similar neurological attributes have also been found in the brains of elephants [79], several species of primates [80] and more recently, in a range of ungulates [81]. Meanwhile, current neurological studies have re-confirmed that VEN cells within the FIC and ACC play a key role in fast-firing social-emotional-autonomic neurological functions, including attributes such as innate, affective empathy [82]. Assuming that analogous neuroanatomical form and location would be associated with analogous function, potentially a range of animals, including humpback whales, may be physiologically equipped to express affective empathy, and this could be associated with epimeletic behavior. At this time, confirmation of the function of these cells within animal brains is still an area of active research. However, further research, including the documentation of stress levels in both distressed and companion animals during entanglement events, could serve to confirm, or refute, this possible explanation.

Notwithstanding any potential mechanistic explanation, the persistence of this behavioral response over time suggests that this is a stable behavioral strategy, with associated fitness benefits. Direct fitness benefits would clearly justify the response of maternal females or close relatives to the distress of a related offspring. Of the incidents reported by Hawai‘i and Alaska entanglement response teams, 26 of 62 incidents involved mothers with dependent calves; direct fitness benefits are likely at stake in these cases. However, where epimeletic interactions involve adult conspecifics (11 of 62 incidents), direct fitness benefits are less apparent.

The mating system of humpback whales is polygynous [83], therefore relatedness among any two random adults is minimal. Still, in situations where an entangled whale survives, the strengthening of social bonds between unrelated individuals could carry fitness benefits. While most social affiliations among humpback whales, on both breeding and feeding grounds, are short-lived [84,85], long-term social associations between mature pairs have been documented. Benefits associated with these longer associations include increased foraging efficiency, through inclusion in co-operative foraging activities [86,87]: Inclusion in group protection during predation events would also carry fitness benefits [88], and future mating opportunities could also be enhanced. In other species studied to date, fitness benefits associated with epimeletic behavior include the cessation of directed aggression (orangutans, *Pongo pygmaeus wurmbii* [89]), increased vigilance of juveniles, and removal of foreign objects (elephants, *Loxodonta africana*. [90]) and increased group cohesion (wolves, *Canis lupus* [91]). Using resources such as Happywhale, further research examining the life histories of whales involved in epimeletic behavior might reveal on-going associations, and future fitness-based benefits, derived through these associations.

The epimeletic response of one whale to an unrelated conspecific could also be interpreted as an example of altruism. In its simplest form, altruistic behavior benefits the recipient at the cost of the donor [54]. Whether altruism exists in the animal world remains an issue of debate. Both classic and more recent theoretical reviews propose that kin selection and associated indirect fitness benefits, even if distant, serve as the underlying justification for seemingly altruistic responses among adult individuals [92,93]. For humpback whales, this explanation has been applied to explain mobbing behavior in response to predatory attacks by transient (Bigg’s) killer whales [88]. Operating across the North Pacific within a basin-wide population structure, Pitman et al. [88] propose that distant kin selection and future reciprocal altruism offset the potential costs of injury when humpback whales intrude during predatory attacks by killer whales. However, the events reported by Pitman et al [88] include instances where humpback whales intervene during killer whale predatory attacks targeting other species, such as harbor seals, (*Phoca vitulina*), California sea lions (*Zalophus californianus*) and ocean sunfish (*Mola mola*). In these instances, as the authors note, interspecies altruism cannot be ruled out.

In the observations compiled here, companion whales drove away assemblages of tiger sharks, supported conspecifics at the surface and ensnared the lines of whaling crews. These actions could increase the survivability of an incapacitated whale. Scarring studies conducted to date [16,17] indicate that although the majority of whales will experience an entanglement at some point in their lives, many will free themselves, and whales that eventually succumb to entanglement injuries may survive for periods of up to six months following the original entanglement [14]. If the presence of other whales during periods of entanglement could offset, reduce, or at least delay the risks of predation or drowning during this highly vulnerable period, then the potential costs for the companion whale could be offset by the benefits of future reciprocal support, when they, in turn, face the challenges of an entanglement event.

Today, the risk of entanglement is just one of many threats facing large whales [6,94]. It is undoubtedly poignant to consider that recent observations of epimeletic behavior among humpback whales draw a direct connection between the decimation of whaling stocks in previous times and the threats posed by the ever-increasing commercial fishing effort, mooring lines and marine debris of the modern era. [6,94]. On a positive note, the on-going innovation and dedication that characterizes the work done by the current network of large whale disentanglement rapid response teams continues to translate into increasing success rates and ever-improving outcomes for whales that become entangled [18]. Further research examining the levels of stress involved, the impact of these events on long term survivability and fecundity, and the ways in which the current generation of whales may be adapting to this modern-day threat, will all contribute towards a better understanding of these events. Such findings can then be applied to improve long term outcomes, enhance the safety and success of entanglement response teams, and hopefully make our oceans less treacherous for the next generation of great whales.

## Supporting information

S1 Table Observations during 62 entanglement events observed in Hawaiian and Alaskan waters between 2001 and 2023. (PDF)
