# Supplementary material for: Tending the sick: Observations of epimeletic behavior in humpback whales towards conspecifics during entanglement events
Source: PLoS One. 2025 Apr 15;20(4):e0321284. doi: 10.1371/journal.pone.0321284 (PMC11999166; doi:10.1371/journal.pone.0321284)
Supplement: S1 Table — (PDF) [file pone.0321284.s001.pdf]

Appendix S1: Table S1: Observations during entanglement events observed in Hawaiian and Alaskan waters between 2001 and 2023

Date	State	Group	Age Class (Entangled)	Observed behavior	Notes
Adult groups					
2/6/2007	HI	Mul	Adult	Associated	No details provided
2/23/2007	HI	Mul	Juvenile	Associated	No details provided
3/2/2007	HI	Mul	Adult	Standing by	Some blocking behavior
2/10/2008	HI	Mul	Adult	Associated	Two other adult whales with entangled whale
8/14/2008	AK	Mul	Adult	Associated	Entangled whale swimming with two others
12/13/2008	HI	Mul	Adult	Associated	Three travelling closely together, one entangled
1/13/2009	HI	Mul	Juvenile	Associated	Sub adult entangled whale, with 3 adults
1/19/2009	HI	Mul	Adult	Associated	Entangled whale swimming with others
1/18/2011	HI	Dyad	Juvenile	Associated	Entangled whale associated with another whale
1/28/2011	HI	Dyad	Adult	Associated	Entangled whale associated with another whale
8/30/2012	AK	Mul	Adult	Associated	Entangled whale in group
3/8/2013	HI	Mul	Juvenile	Standing by	Entangled whale joined adult group. Group provided protection during response efforts
1/10/2014	HI	Dyad	Adult	Associated	Entangled whale associated with another whale.
2/14/2014	HI	Dyad	Adult	Associated	One adult travelling with entangled whale
4/6/2014	HI	Dyad	Juvenile	Associated	Entangled whale swimming with others
2/13/2015	HI	Mul	Adult	Assisting	Some blocking behaviors from one whale, protecting entangled whale
3/16/2015	HI	Mul	Adult	Associated	Entangled whale swimming with others
9/7/2015	AK	Dyad	Adult	Associated	Entangled whale swimming with others
5/16/2016	AK	Mul	Adult	Associated	Entangled whale swimming with others
6/4/2016	AK	Mul	Adult	Standing by	Entangled whale, accompanied by second whale, swimming together along shoreline. Multiple resights together
1/4/2018	HI	Dyad	Adult	Associated	Entangled whale part of adult group, group splintered, entangled whale left as part of a Dyad
1/11/2018	HI	Dyad	Adult	Assisting	Second whale seen circling a stationary entangled whale.
1/21/2018	HI	Dyad	Juvenile	Associated	Associated with another whale.
1/27/2018	HI	Dyad	Adult	Standing by	Second whale stayed beside entangled whale throughout disentanglement operations
2/4/2018	HI	Dyad	Juvenile	Associated	Second whale remained in association with entangled whales during initial response
2/13/2018	HI	Mul	Adult	Associated	Associated with multiple adults
2/17/2018	HI	Dyad	Adult	Associated	Entangled whale swimming with another whale
5/20/2018	HI	Dyad	Adult	Associated	Entangled whale swimming with second whale
2/17/2019	HI	Dyad	Adult	Standing by	Second whale joined entangled whale during response efforts
6/28/2020	AK	Dyad	Adult	Standing by	Entangled whale making shallow dives close to shore. Second adult moving in tandem
8/18/2020	AK	MC	Juvenile	Standing by	Entangled whale, ~8m accompanied by larger whale
2/13/2021	HI	Mul	Juvenile	Supporting	Entangled whale alone when first sighted. Resighted 3/9, 3/10, second whale closely associated, fending off sharks, supporting entangled whale at surface. Resighted 3/11 alone.
2/4/2022	HI	Dyad	Adult	Associated	Entangled whale accompanied by another adult.

2/14/2022	HI	MCE	Adult	Standing by	Mother entangled; escort stayed with her.
3/5/2022	HI	MCE	Adult	Associated	Entangled whale swimming with MC pair
12/30/2023	HI	Dyad	Juvenile	Associated	Two adult whales traveling together, one entangled
Calf groups					
6/15/2001	AK	MC	Mother + calf	Associated	Entangled calf trailing lines from rostrum forward of blowhole, same line visible on mothers back
7/1/2007	AK	MC	calf	Standing by	Entangled calf, mother stayed close by, calf eventually freed itself
1/2/2008	HI	MC	Calf	Associated	No details provided
4/26/2008	HI	MC	Calf	Standing by	Entangled calf, mother stayed close by
7/14/2009	AK	MC	Calf	Associated	Entangled calf accompanied by mother
9/12/2009	AK	MC	Mother	Associated	Mother entangled, calf staying close by
12/1/2009	HI	MCE	yearling	Standing by	Escort displayed block behavior and close passes to any response approaches
6/22/2010	AK	MC	Mother + calf	Assisting	Entangled calf had gillnet mesh over its back and dorsal fin, travelling with mother who has same filament around fins
7/27/2010	AK	MC	Mother + calf	Assisting	Mother and calf entangled together, self-disentangled, seen free swimming afterwards.
12/16/2010	HI	MC	Mother	Associated	Mother entangled, calf following close by
3/30/2011	HI	MC	Mother	Associated	Mother entangled
10/4/2011	AK	MC	Mother	Associated	Mother entangled, calf stayed nearby
5/1/2012	AK	MC	Calf	Standing by	Entangled calf, mother stayed by calf, calf eventually freed
2/28/2013	HI	MC-EE	Calf	Assisting	Entangled calf, protective mother and escorts
12/15/2013	HI	MCE	Calf	Assisting	Entangled calf, moving in coordination with mother and escort, attending and blocking approaches during disentanglement efforts
3/23/2015	HI	MC	Calf	Associated	No details provided
2/6/2016	HI	MC	Mother	Associated	Mother entangled around flukes, calf close by
6/30/2016	AK	MC	Calf	Associated	Mother present, calf entangled
7/11/2016	AK	MC	Calf	Standing by	Calf entangled, mother stayed in area
5/13/2019	AK	MC	Calf	Standing by	Mother stayed close beside entangled calf
7/26/2020	AK	MC	Calf	Mother left	Calf entangled. Mother not seen for 20 minutes. Calf freed by nearby vessel, and MC subsequently seen together
1/13/2021	HI	MC	Mother	Associated	No details provided
8/26/2021	AK	MC	Calf	Standing by	Calf trailing several feet of gill net. Mother stayed with calf. Seen again on 8/30/21, still together
2/12/2022	HI	MCE	Mother	Standing by	Mother entangled, calf and escort stayed with her. Resighted 2/14, both still present
11/1/2022	HI	MC	Mother	Associated	No details provided
7/17/2023	AK	MC	Calf	Standing by	Calf successfully disentangled; mother stayed close by throughout.
